# microRNA-141 inhibits TGF-β1-induced epithelial-to-mesenchymal transition through inhibition of the TGF-β1/SMAD2 signalling pathway in endometriosis

**DOI:** 10.1007/s00404-019-05429-w

**Published:** 2020-01-04

**Authors:** Sixue Wang, Mengmeng Zhang, Tingting Zhang, Juan Deng, Xiaomeng Xia, Xiaoling Fang

**Affiliations:** Department of Obstetrics and Gynecology, The Second Xiangya Hospital, Central South University, No. 139 Renmin Road, Changsha, 410000 Hunan P.R. China

**Keywords:** Endometriosis, EMT, miR-141, TGF-β1/SMAD2 signalling pathway

## Abstract

**Purpose:**

Recent studies have demonstrated the differential expression of micro(mi)RNAs in endometriosis. Previously, we reported the low expression of miR-141 in patients with this disease. Epithelial-to-mesenchymal transition (EMT) and the transforming growth factor-beta1 (TGF-β1)-induced SMAD2 signalling pathway are central to tumour proliferation and invasion. However, the role of miR-141 in regulating the TGF-β1/SMAD2 signalling pathway and the associated EMT to be elucidated.

**Methods:**

The levels of TGF-β1/SMAD2 signalling and EMT markers expression in eutopic and ectopic endometria of endometriosis were determined by immunohistochemistry and western blot analyses. MiR-141 expression was analysed by quantitative reverse-transcription polymerase chain reaction. Cellular invasion and proliferation were determined by transwell and CCK-8 assays, respectively. Functional assay of miR-141 was performed using plasmid and shRNA transfection methods.

**Result:**

The presence of miR-141, EMT, and TGF-β1/SMAD2 signalling markers were detected in eutopic and ectopic endometria of endometriosis. TGF-β1-induced EMT in Ishikawa (ISK) cells by activating the SMAD2 signalling pathway, whereas miR-141 inhibited the TGF-β1-induced EMT, proliferation and invasion abilities of these cells.

**Conclusion:**

These data identify miR-141 as a novel driver of EMT in endometriosis, implicates the link between miR-141 and TGF-β1/SMAD2 signalling pathway in the context of endometriosis, and underscore the role of EMT in the development of endometriosis.

## Introduction

Endometriosis is a condition in which endometrial cells grow outside the uterus, causing infertility and pelvic pain in women, affecting 6–10% of women of reproductive age [1, 2]. Although endometriosis is considered benign gynecological disease, it has cancer-like features as it can recapitulate certain features of malignant neoplasms, including migration and resistance to apoptosis [3, 4]. Sampson’s theory of retrograde menstruation is the most widely accepted hypothesis for the aetiology of endometriosis [5, 6]. However, the pathogenesis of this condition is still controversial and has yet to be elucidated.

EMT is a cellular process in which polarised immotile epithelial cells are converted into invasive and migratory mesenchymal cells as a result of their interaction with membrane structures, such as adherens and gap junctions [7, 8]. EMT is considered an essential process associated with cancer metastasis and invasion. EMT can be induced by multiple signals, including estrogen stimulation and TGF-β signalling. TGF-β signalling plays vital roles in embryonic development as well as in cellular homeostasis [9]. TGF-β from the inflammatory tumour microenvironment may induce either tumour cell apoptosis and cancer suppression or induce an EMT to promote tumour cell invasion and metastasis [10, 11]. TGF-β is a principal component of the signalling associated with endometriosis; for instance, the activity of TGF-β1 is increased in the peritoneal fluid of women with this condition [7, 12]. Recent studies suggest that overactive TGF-β1/SMAD2 signalling further contributes to the establishment of an EMT process in advanced breast cancers [13]. Because endometriosis has tumor-like features in terms of cell migration and cell survival, EMT has been postulated to be involved in the mechanism of endometriosis [14]. Considering these findings together, we speculated that TGF-β1/SMAD2 signalling might mediate EMT to promote cellular proliferation and invasion in the pathogenesis of endometriosis.

Emerging studies suggest that abnormal microRNA (miRNA) expression may be implicated in the development of endometriosis [15]. MiRNAs are a class of small non-coding RNAs that bind to the 3′ untranslated region of their corresponding mRNAs and inhibit gene expression by degrading the target mRNA or inhibiting its translation miRNAs play an important role in diverse cellular processes, such as proliferation, invasion, migration, and apoptosis [16, 17]. The miR-200 family members include miR-200a, miR-200b, miR-200c, miR-141, and miR-429, and are key negative regulators of EMT [16, 18, 19]. The miR-200 family members inhibit expression of the related transcriptional repressor zinc finger E-box-binding transcription factor 1 (ZEB1) and ZEB2 [the latter is also known as SMAD-interacting protein 1 (SIP1)] in epithelial cells and play a major role in preventing these factors from triggering EMT [18]. In contrast, TGF-β signalling was shown to be a key inducer of ZEB1 and SIP1 expression and EMT [20]. Recently, some studies showed that TGF-β activity was subject to miRNA control in human tumours, where TGF-β1 and TGF-β2 mRNA translation was repressed by miR-21 [21, 22]. Previously, we had reported down-regulation of miR-141 expression in patients with endometriosis [23]. However, the function of miR-141 in endometriosis remains unknown. In this study, we aimed to elucidate the role of TGF-β1/SMAD2 signalling in the miR-141-mediated effects of growth and metastasis of endometriosis.

## Materials and methods

### Human endometrial tissue samples

In total, 32 patients admitted to the Second Xiangya Hospital of Central South University, who underwent laparoscopy for pelvic endometriosis, were enrolled in the present study. The numbers and characteristics of the endometriotic tissue samples were analysed as follows: 2 endometria of superficial peritoneal endometriosis, 10 pairs of eutopic and ectopic endometria of ovarian endometriosis patients with sexual intercourse, the other 19 ectopic endometria of ovarian endometriosis patients without sexual intercourse, 1 endometrium of deep infiltrating endometriosis. On the basis of the revised American Fertility Social classification, 5 patients had stage I–II endometriosis (2 patients from superficial peritoneal endometriosis group and 3 patients from ovarian endometriosis group) and 27 patients had stage III–IV (1 patient from deep infiltrating endometriosis group and 26 patients from ovarian endometriosis group). As normal controls, surgical samples of normal endometrial tissue were obtained from 17 patients: 8 patients with cervical intraperitoneal neoplasia III, 5 patients with a previous caesarean scar defect, and 4 patients with infertility. None of the women enrolled in the study had received hormone therapy or sex steroids or had used intrauterine contraceptive devices for at least 6 months prior to the surgery. The samples were confirmed histologically according to the established criteria. This study was approved by institutional ethics review board of The Second Xiangya Hospital, Central South University (#2016243). All tissue samples were obtained with full and informed patient consent, and all experiments were followed the ethical principles outlined by the 1964 Helsinki Declaration and its later amendments or comparable ethical standards.

### Immunohistochemistry

Immunohistochemistry (IHC) was performed on 31 pairs of paraffin-embedded tissue sections as described previously [14]. The primary antibodies used in the present study were rabbit polyclonal anti-E-cadherin (1:800; Proteintech, USA), anti-vimentin (1:1000; Proteintech), anti-TGF-β1 (1:300; Proteintech), and rabbit monoclonal anti-SMAD2 (1:50; Abcam, USA). In brief, the paraffin sections were subjected to heat-induced antigen retrieval and then incubated overnight with the respective primary antibody at 4 °C. After rinsing, the tissue sections were incubated with horseradish peroxidase (HRP)-conjugated secondary antibody. The stained specific antigens were visualised using a 3, 3′-diaminobenzidine substrate kit according to the manufacturer’s instructions. Images were captured and analysed using an optical microscope. The intensity of immunohistochemical staining was evaluated using a semi-quantitative grading system (*H* score).

### Cell culture and treatment

ISK cells obtained from the Cell Bank of Advanced Research Center (Changsha, China) were cultured in Dulbecco’s modified Eagle’s medium (Gibco, USA) supplemented with 10% foetal bovine serum (FBS) (Gibco), 50 U/mL penicillin, and 50 U/mL streptomycin at 37 °C under 5% CO_2_. Cells were seeded at a density of 3 × 10^5^ cells/well in 6-well plates and incubated with TGF-β1 (10 ng/mL, Proteintech) and the TGF-β1 receptor inhibitor SB431542 (10 μM, Apexbio, USA) for 24 h.

### Cell invasion and proliferation assays

The cell invasion assay was performed using a 24-well transwell chamber with an 8 μm pore size insert. The upper chamber was covered with Matrigel (Corning, USA) and incubated at 37 °C overnight to allow gelling. Cells (4 × 10^4^) suspended in 250 μL of serum-free culture medium were then seeded onto the upper chamber and 750 μL of culture medium containing 10% FBS was added to the lower chamber and the apparatus was incubated for 24 h. After incubation, cells that had migrated into the lower surface of the membrane were fixed with methanol and stained with crystal violet. Images of three randomly selected fields of the fixed cells were captured and cells were counted under a 20 × objective lens and imaged using ImageJ software. The cell counting kit (CCK)-8 assay (Bimake, USA) was used to determine cell proliferation. In brief, ISK cells were seeded at a density of 6 × 10^3^ cells/300 μL into each well of a 96-well plate and incubated for 0, 12, and 24 h, and then processed further according to the manufacturer’s protocol. The optical density of the ISK cells cultured under the different culture conditions was then measured.

### RNA extraction and quantitative reverse-transcription polymerase chain reaction

Total RNA was extracted from the human tissues using Trigol reagent (DingGuo, China). The first-strand cDNA was synthesised from 4 μg of total RNA using the Mir-X™ miRNA First-Strand Synthesis Kit (Clontech, Japan) according to the manufacturer’s protocol. The quantitative reverse-transcription polymerase chain reaction (qRT-PCR) for miR-141 was performed using the Mir-X™ miRNA qRT-PCR TB Green Kit (Clontech, Japan) with LightCycler 96 software according to the manufacturer’s protocol. The primer pair used for qRT-PCR was 5′-TAACACTGTCTGGTAAAGATGG-3′ (TSINGKE, China). The PCR thermal conditions were as follows: initial denaturation at 95 °C for 30 s, followed by 40 cycles of 95 °C for 5 s, 60 °C for 20 s, 95 °C for 1 s, 65 °C for 15 s, and 95 °C for 1 s for the dissociation curve.

### Western blot analysis

Human tissue samples and cultured cells were lysed in radio-immunoprecipitation assay buffer (Beyotime, China) for protein extraction. In brief, 15–25 μg of the protein was first resolved by 10% sodium dodecyl sulphate polyacrylamide gel electrophoresis, and the bands were then electro-blotted onto polyvinylidene difluoride membranes (Millipore, USA). The membranes were blocked with 5% skim milk or 5% bovine serum albumin for 1 h and then incubated overnight at 4 °C with the following primary antibodies: rabbit polyclonal anti-E-cadherin (1:2500), anti-vimentin (1:2000), anti-TGF-β1 (1:1000), rabbit monoclonal anti-SMAD2 (1:5000), rabbit monoclonal anti-phospho-SMAD2 (1:1000, Cell Signalling Technology, USA), and anti-GAPDH (1:20,000, Proteintech). Following this, the membranes were washed three times with phosphate-buffered saline Tween-20 (PBS-T) and incubated with HRP-conjugated AffiniPure goat anti-rabbit IgG (1:5000, Proteintech). Signals were visualised using the enhanced chemiluminescence reagent according to the manufacturer’s protocol.

### Cell transfection with plasmids and small hairpin RNA

A small hairpin RNA (shRNA) against miR-141, and miR-141 overexpression (i.e. miR-141 mimic) and negative control plasmids were designed and synthesised by Vector Builder (Guangzhou, China). The shRNA or the respective plasmids were transfected into ISK cells using Lipofectamine 2000 (Life Technologies, USA) according to the manufacturer’s instructions. In brief, the cells were first seeded at a density of 4 × 10^4^ cells/well in a six-well plate and grown until 60–80% confluence. The cells were then cultured in serum and antibiotic-free medium for 30–60 min and thereafter transfected with the miR-141 mimic or miR-141 shRNA or their respective control plasmids according to the manufacturer’s instructions.

### Immunofluorescence assay

The ISK cells were fixed with 4% paraformaldehyde and permeabilised with Triton-X100 (Sigma, USA). The sections were incubated with p-SMAD2 (1:50, Cell Signalling Technology, USA) at 4 °C overnight, followed by Alexa Fluor 594-conjugated goat anti-rabbit IgG (H + L) antibody (1:1000, Proteintech). Finally, 4′, 6-diamidino-2-phenylindole solution (Wellbio, China) was used to stain the cell nuclei.

### Statistical analysis

Comparisons between two measurements were determined using Student’s *t* test. The groups were analysed using one-way analysis of variance. All statistical analyses were performed using SPSS software and GraphPad Prism 7.0 software. *P* values of < 0.05 were considered significant.

## Results

### Abnormal EMT and TGF-β1/SMAD2 signalling pathway-related molecules expression were observed in endometriosis

**Fig. 1:**
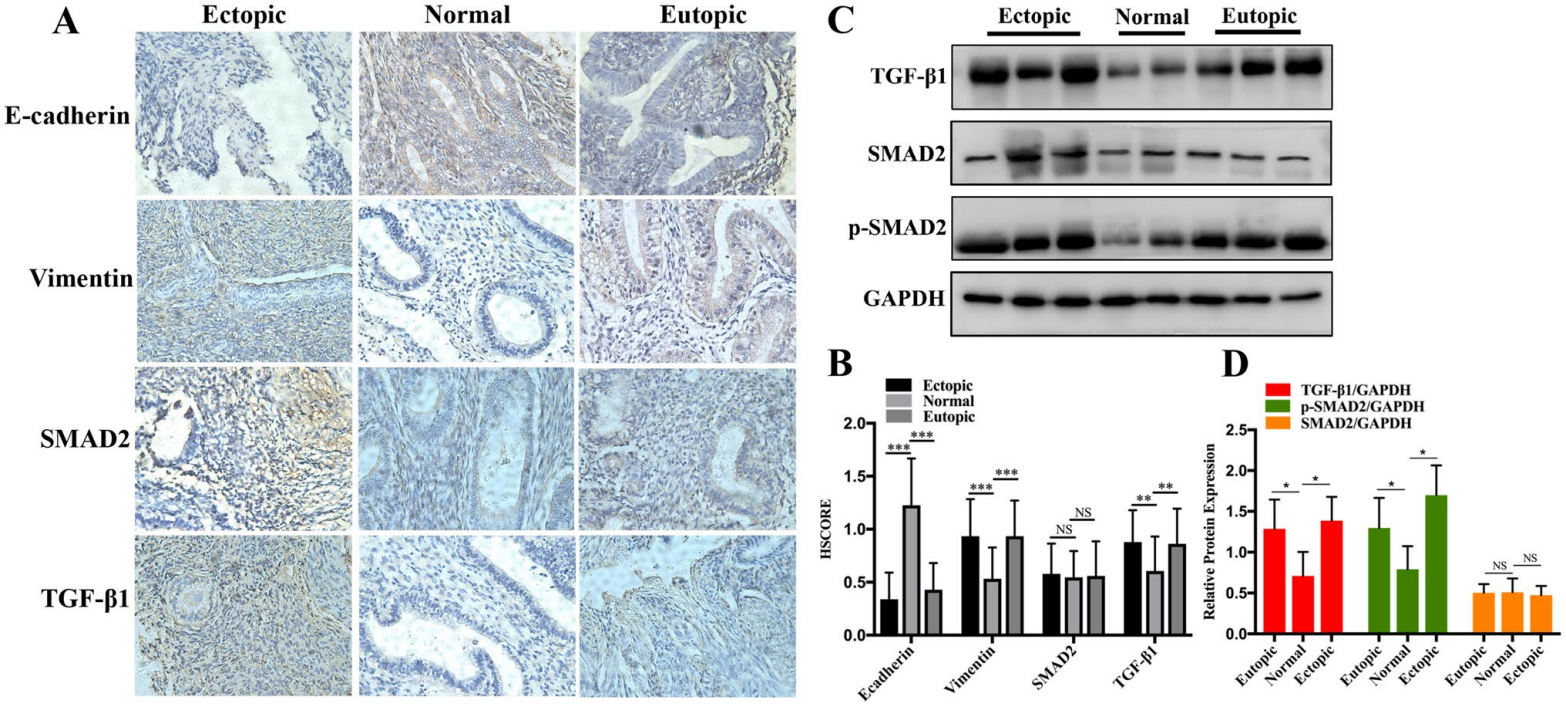
Abnormal EMT and TGF-β1/SMAD2 signalling pathway-related molecule expression were observed in endometriosis. **a**, **b** Immunohistochemical analysis of the expression of TGF-β1, SMAD2, vimentin and E-cadherin in ectopic and eutopic endometria isolated from endometriosis and normal endometrial tissue, respectively. HSCORES are expressed as mean ± SEM. **c**, **d** The protein expression levels of TGF-β1, SMAD2, p-SMAD2, vimentin and E-cadherin among the three tissue samples were determined by western blot analysis. *P < 0.05, **P < 0.01, ***P < 0.001 and *NS* non-significant

To investigate the involvement of EMT and TGF-β1/SMAD2 signalling pathway in the development of endometriosis, IHC and western blot analyses of E-cadherin, vimentin, TGF-β1, SMAD2, and phospho-SMAD2 expression were performed on eutopic and ectopic endometria of endometriosis and on normal endometrial tissue. Compared with the normal endometrial tissues, the expression of vimentin and TGF-β1 were positive stained in the majority of eutopic and ectopic endometrial tissues detected by IHC. In contrast, the expression of E-cadherin was significantly lower compared with the normal tissues. However, there was no significant difference in the positive staining rate of SMAD2 between eutopic and ectopic endometria of endometriosis and normal endometrial tissue (Fig. 1a, b). Furthermore, we analysed the protein expression profile of TGF-β1/SMAD2 signalling pathway in the three types of endometrial tissues. The expression of TGF-β1 and p-SMAD2 was significantly increased in the endometriotic samples compared with that in the normal sample. However, the total SMAD2 expression levels were not significantly different among the three tissue samples (Fig. 1c, d). Taken together, these results support the occurrence of EMT during endometriosis. The TGF-β1/SMAD2 signalling pathway could be speculated to be involved in the pathological process of endometriosis.

### TGF-β1 promotes EMT in ISK cells by activating the SMAD2 signalling pathway

**Fig. 2:**
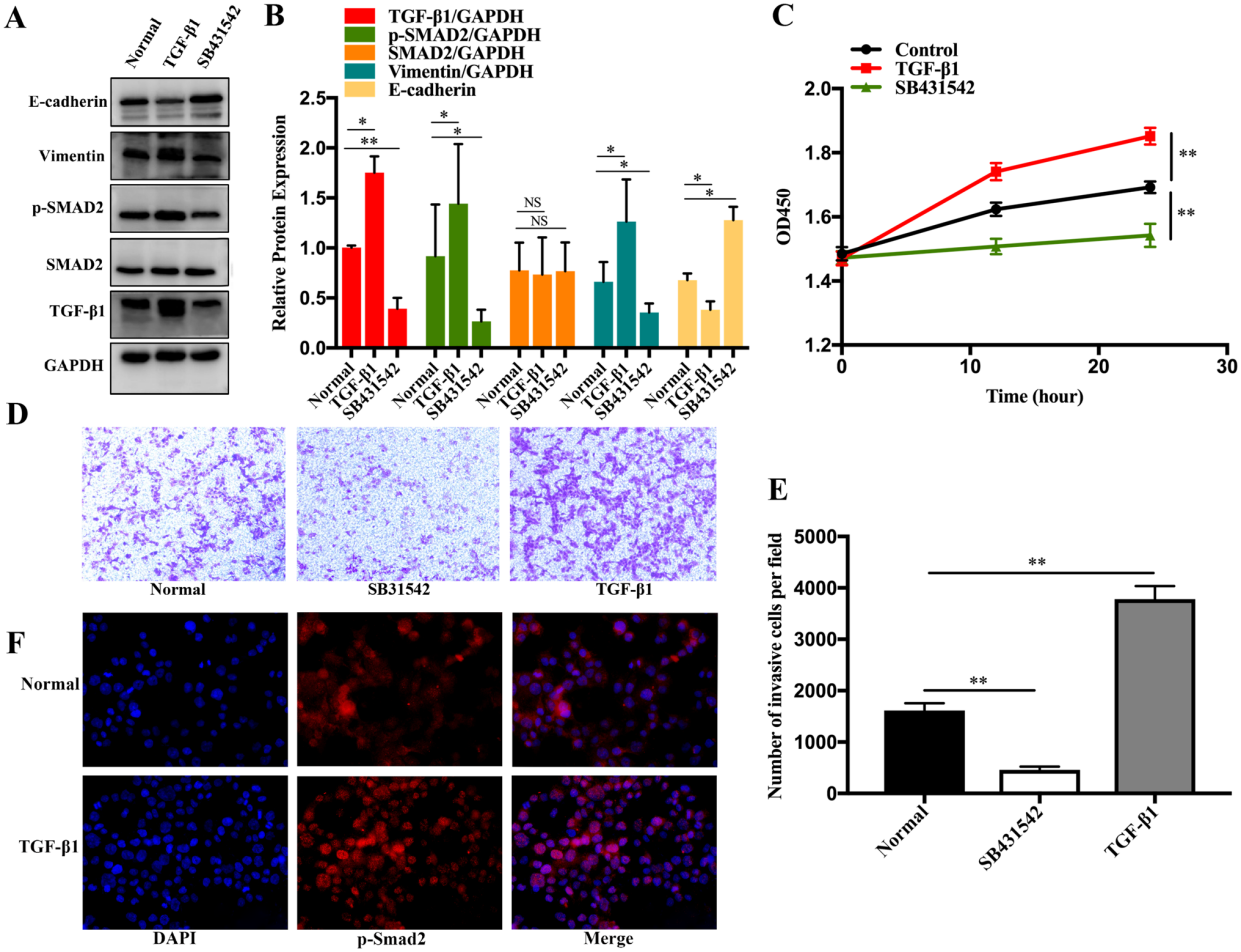
TGF-β1 promotes EMT in ISK cells by activating the SMAD2 signalling pathway. **a**, **b** Western blot analysis of protein expression after ISK cells treatment with recombinant TGF-β1 or the TGF-β1 inhibitor SB431542 for 24 h. **c** CCK-8 assay of the viability of ISK cells subjected to different treatments. **d**, **e** Transwell assay to evaluate the invasion of ISK cells treated with recombinant TGF-β1 or SB431542 for 24 h. **f** The immunofluorescence assay revealed the location of p-SMAD2 in ISK cells treated with recombinant TGF-β1. *P < 0.05, **P < 0.01 and *NS* non-significant

To investigate the interaction between TGF-β1/SMAD2 signalling and EMT, ISK cells were incubated with recombinant TGF-β1 or the TGF-β1 inhibitor SB431542 for 24 h, and then, total protein was extracted for western blot detection of TGF-β1/SMAD2 signalling and epithelial marker (E-cadherin) and mesenchymal marker (vimentin) expression. Phenotypic analyses of the cells were based on invasion and adhesion assays. Recombinant TGF-β1 significantly enhanced the expression of TGF-β1, p-SMAD2, and vimentin and decreased the expression of E-cadherin, without altering the total SMAD2 levels (Fig. 2a, b). In contrast, SB431542 inhibited the recombinant TGF-β1-stimulated enhancement of expression of these proteins (Fig. 2a, b). The CCK-8 and transwell assays indicated enhanced cell invasion and proliferation of TGF-β1-stimulated ISK cells compared with that of control cells (Fig. 2c–e). p-SMAD2 is localised mainly in the cytoplasm of embryonic stem cells. However, immunofluorescence assay revealed the localisation of p-SMAD2 in the nucleus of the recombinant TGF-β1-treated ISK cells in this study (Fig. 2f). Taken together, these results suggest that the induction of EMT to promote proliferation and invasion of ISK cells occurs through activation of the TGF-β1/SMAD2 signalling pathway.

### miR-141 is down-regulated in endometriosis

**Fig. 3:**
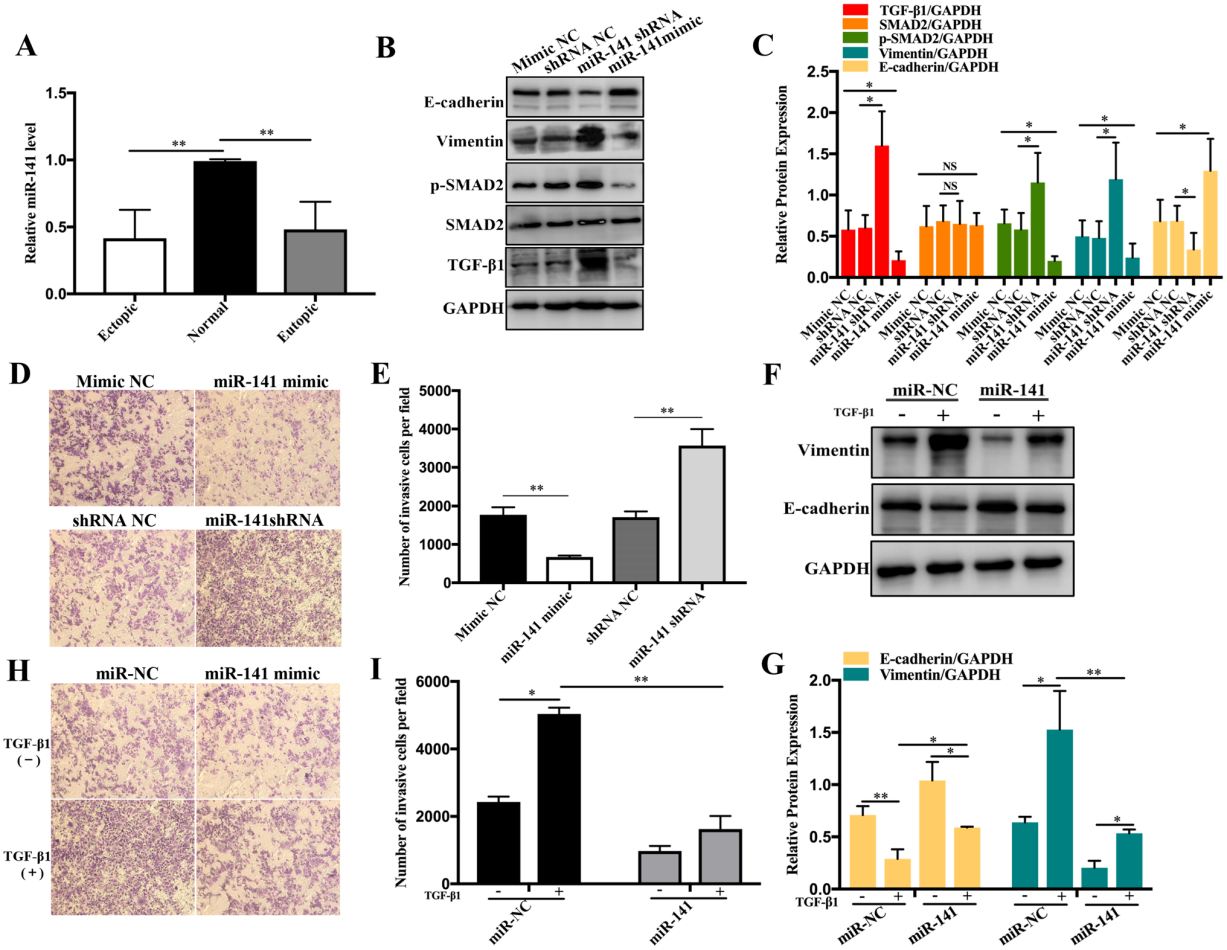
Expression of miR-141 is down-regulated in eutopic and ectopic endometria and miR-141 overexpression inhibited the TGF-β1-induced EMT and invasion abilities of ISK cells in vitro. **a** miR-141 was detected among the three tissue samples by qRT-PCR analysis. **b**, **c** The TGF-β1, SMAD2, p-SMAD2, vimentin and E-cadherin protein expression levels were determined by western blot analysis in ISK cells transfected with the miR-141 mimic (2 µg/mL) or miR-141 shRNA (2 µg/mL) for 24 h. **d**, **e** Phenotypic analyses of cells transfected with the miR-141 mimic or miR-141 shRNA by transwell assay. The numbers of migrated cells were counted in the microscope fields. **f**, **g** ISK cells were transfected with miR-141 or miR-NC (2 µg/mL) and then treated with or without TGF-β1 (10 ng/mL) for 24 h. The protein expression levels of E-cadherin and vimentin were determined by western blot assay. **h**, **i** Phenotypic analyses of endometrial adenocarcinoma cells by transwell assay. The cells transfected with miR-141 or miR-NC were stimulated with TGF-β1 (10 ng/mL) for 24 h. The numbers of migrated cells were then counted in the microscope fields. *P < 0.05, **P < 0.01 and *NS* non-significant

The expression of miR-141 in ectopic and eutopic endometria and in normal endometrial tissue was analysed by qRT-PCR. MiR-141 was significantly down-regulated in the tissues with eutopic and ectopic endometria compared with that in the normal endometrial tissue (Fig. 3a). Because TGF-β1 could induce EMT by activating the SMAD2 signalling pathway, and given that miR-141 is down-regulated in endometriosis, we further investigated the possible influence of miR-141 on TGF-β1-induced EMT.

### miR-141 inhibits the TGF-β1-induced EMT and invasion of endometrial adenocarcinoma cells

To gain insight into the mechanisms underlying the function of miR-141 in endometrial cells, the expression of the TGF-β1/SMAD2 signalling pathway and EMT markers (i.e. TGF-β1, p-SMAD2, SMAD2, E-cadherin, and vimentin) in ISK cells transfected with plasmids carrying an miR-141 mimic or an miR-141 shRNA was analysed. The western blot results indicated the significantly decreased expression of TGF-β1, p-SMAD2, and vimentin, and increased expression of E-cadherin in the presence of the miR-141 mimic (Fig. 3b, c). Furthermore, the invasion abilities of the ISK cells were investigated and found to be lower in the miR-141 mimic group than in the miR-141 shRNA group (Fig. 3d, e). We had found that TGF-β1 markedly promoted the expression of vimentin and suppressed the expression of E-cadherin in ISK cells. However, miR-141 overexpression attenuated both the recombinant TGF-β1-mediated inhibition of E-cadherin expression and enhanced expression of vimentin (Fig. 3f, g). In addition, miR-141 overexpression inhibited the TGF-β1-induced invasion of ISK cells. Phenotypic analyses of the endometrial adenocarcinoma cells by transwell assay confirmed this inhibitory effect of miR-141 on TGF-β1-induced EMT (Fig. 3h, i). Taken together, our results indicated that miR-141 could suppress TGF-β1-induced EMT and cell invasion by inhibiting the TGF-β1/SMAD2 signalling pathway.

## Discussion

Recently, a meta-analysis evaluated the associations between endometriosis and risk and prognosis of ovarian cancer in studies published between 1990 and 2012. A significant positive association was observed regardless of study design, assessment of endometriosis, quality score of study or number of adjustment factors [3, 5]. Thus, identifying new biomarkers for early diagnosis as well as detection of specific therapeutic targets for the treatment of endometriosis is urgently needed [1, 2]. EMT is activated upon interaction with extracellular signals, including components of the extracellular matrix and soluble growth factors, such as members of the TGF-β family [12, 14]. TGF-β-mediated EMT is an important mechanism of neoplastic cell metastasis and invasion [7]. The nuclear import of SMAD2 is mediated by direct interaction with the nucleoporin CAN/Nup214. The transcription factor SMAD2 is released from cytoplasmic retention through its TGF receptor-mediated phosphorylation and accumulates in the nucleus where it associates with co-factors to regulate transcription [24]. Hence, inhibition of TGF-β signalling decreased the cancer-like behaviour of the cells.

In our study, we found the enhanced expression of mesenchymal markers (e.g. vimentin) and decreased expression of epithelial markers (e.g. E-cadherin) in eutopic and ectopic endometria of endometriosis, confirmed the involvement of EMT as an important factor that participated in the endometriosis. Moreover, results of the present study identified that TGF-β1 promoted EMT in ISK cells by activating the SMAD2 signalling. Our results are consistent with the earlier report that TGF-β1/SMAD2 signalling pathway may be involved in EMT.

There is accumulating evidence that various epigenetic aberrations exist in endometriosis**.** Publications, up to the end of June 2019, pertaining to epigenetic aberration in endometriosis were identified through PubMed. Epigenetics appears to be a common denominator for hormonal and immunological aberrations in endometriosis [25]. Various studies on miRNAs in endometriosis have identified their cardinal role in the pathogenesis of the disease, such as the miR-200 family and have taken them as potential biomarkers in endometriosis [21]. As with the eutopic endometrial tissue, its ectopic counterpart responds to cyclic changes in steroid hormones by proliferation differentiation and by the production of autocrine and paracrine factors. As reported by some studies, however, ectopic endometrium appears to behave different from its eutopic counterpart in many other ways [21]. In the present study, we confirmed the down-regulation of miR-141 in endometrial tissues with ectopic and eutopic relative to that in normal endometrial tissue. Although the expression miR-141 was detected in endometriotic tissues, it was not clear whether miR-141 was a necessary protective factor against the endometriosis. The regulation of TGF-β1-induced EMT by miR-141 and the subsequent effect on cell invasion in endometriosis still needed to be elucidated. To understand the function of miR-141 in the progression of endometriosis, its effect on EMT was analysed. Our studies show that miR-141 inhibited the TGF-β1-induced EMT and invasion abilities of ISK cells. Moreover, inhibition of miR-141 enhanced the activity of TGF-β in endometriosis. The reduced invasiveness of miR-141-transfected cells shown by this study proved the role of miR-141 as a potential protective factor against the development of endometriosis.

This study also has several limitations. First, we used ISK cells to instead the endometrial epithelial cells for transfection and the loss-of-function experiments. Although ISK cells, a well-differentiated human endometrial adenocarcinoma cell line, bears estrogen and progesterone receptors. Moreover, ISK cells retain the phenotype of endometrial epithelial cells and display a similar expression profile of molecules to endometrium regulated by estrogen and progesterone. It also has similar apical adhesiveness ability with normal endometrial cells [26, 27]. Hence, ISK cells have been used for numerous basic research areas such as reproductive biology and molecular science including endometriosis researches [28, 29]. Second, further in vivo studies with appropriate animal models are required to verify these in vitro findings. Third, we did not evaluate the possible correlation of miR-141 with the transcription factors, such as ZEB1 and ZEB2. Hence, the precise role of miR-141 in regulating EMT process still remain to be investigated.

In summary, the present study proves that miR-141 is an inhibitor of the TGF-β1/SMAD2 signalling pathway, and results in the inhibition of TGF-β1-induced EMT in endometriosis. Our results provide a potential rationale for the role of miR-141 in the cellular invasion and proliferation associated with the progression of endometriosis. Thus, miR-141 could be used as a potential molecular diagnostic tool to detect endometriosis in women.

## Conclusion

We identified miR-141 as a novel inhibitor of the TGF-β1/SMAD2 signalling pathway and subsequently of EMT in endometriosis. Our findings help to understand the etiology of cancer-like features of endometriosis and provide a molecular framework that should be beneficial for the design of new therapeutic strategies to prevent the recurrence of disease.
